# Complementary utility of plasma biomarkers and Aβ‐PET for diagnosis, risk‐stratification, and treatment monitoring in Alzheimer's disease

**DOI:** 10.1002/alz.70763

**Published:** 2025-10-14

**Authors:** Lyduine E. Collij, Niklas Mattsson‐Carlgren, Shorena Janelidze, Rik Ossenkoppele, Oskar Hansson

**Affiliations:** ^1^ Clinical Memory Research Unit Department of Clinical Sciences Malmö Faculty of Medicine Lund University Lund Sweden; ^2^ Radiology and Nuclear Medicine Amsterdam UMC, location VUmc Amsterdam the Netherlands; ^3^ Brain Imaging Amsterdam Neuroscience Amsterdam the Netherlands; ^4^ Memory Clinic Skåne University Hospital Malmö Sweden; ^5^ Wallenberg Center for Molecular Medicine Lund University Lund Sweden; ^6^ Neurology, Alzheimer center Amsterdam Amsterdam UMC, location VUmc Amsterdam the Netherlands; ^7^ Neurodegeneration Amsterdam Neuroscience Amsterdam the Netherlands

**Keywords:** amyloid‐PET, blood biomarkers, clinical routine, clinical trials

## Abstract

With the rapid development of blood biomarkers (BBMs) related to amyloid‐β (Aβ) pathology in Alzheimer's disease (AD), the question arises whether these can replace the accepted reference standard, positron emission tomography (PET), for assessing Aβ burden. BBMs can differentiate Aβ status reliably in cognitively impaired patients, but two‐threshold strategies to further improve their performance demonstrate the need for pathological confirmation by Aβ‐PET in a non‐negligible portion of individuals (∼10%–40%), especially in early‐stage disease where BBM performance declines due to lower AD prevalence, reducing the test's positive predictive value (PPV) and increasing the risk for false‐positives. This may be increasingly relevant in the future, considering the development of (very) early interventions against AD. Further, BBMs do not accurately reflect the actual Aβ load or change after immunotherapy. Consequently, there are clear remaining needs for Aβ‐PET in several clinically important settings as (i) a confirmatory test and (ii) to determine treatment response.

**Highlights:**

- When used as stand‐alone tests, blood biomarkers (BBMs) demonstrate good sensitivity and specificity (84%–90%) relative to amyloid‐β positron emission tomography (Aβ‐PET).
- Two‐threshold strategies improve BBM performance but require confirmatory testing by, for example, Aβ‐PET in a non‐negligible portion of patients that fall in an intermediate range or “gray‐zone” (∼10%–40%).
- In early/preclinical populations, BBM performance declines due to lower AD prevalence, reducing the test's positive predictive value (PPV) and increases the gray‐zone population.
- Currently available BBMs cannot reliably estimate Aβ‐PET burden or track Aβ‐plaque removal post‐immunotherapy.

## 1 INTRODUCTION

Dementia is a major cause of disability, dependency, and mortality in the elderly population, with projections estimating up to 150 million individuals affected by 2050.^1^ Alzheimer's disease (AD), the leading cause of dementia, is a neurodegenerative disorder pathologically characterized by amyloid‐β (Aβ) and tau protein accumulation.^2^ The recent approval of several amyloid‐targeting treatments (ATT) has emphasized the need for accurate diagnosis to ensure that treatment is given to patients most likely to benefit from it. However, misdiagnosis of AD is common when using clinical examination alone, ranging from 25%–30% in specialized clinics^2^ up to more than 40%–50% in primary care.^3^

The arrival and subsequent global regulatory approvals of Aβ positron emission tomography (PET) tracers in 2004 and onward revolutionized the dementia field, allowing accurate in vivo assessment of the extent and deposition pattern of Aβ pathology.^4^ Today, biomarker confirmation is an essential component of AD diagnosis.^5^, ^6^, ^7^ Although associated with high clinical utility,^8^, ^9^, ^10^ the use of PET is hampered by higher costs, radiation exposure, and limited accessibility, hence limiting larger‐scale clinical use. The first emergence of blood biomarkers (BBMs) has paved the way toward more easily accessible assessment of Aβ pathology.^11^, ^12^, ^13^, ^14^ The rapid development in BBMs have led to their ongoing implementation in clinical practice and trial settings,^3^, ^15^, ^16^, ^17^ with the first United States Food and Drug Administration (FDA) approved blood test to aid in the diagnosis of Alzheimer's disease in May 2025.^18^

This perspective of the current literature highlights the advancements brought by BBMs, with the aim to clarify in which settings BBMs are most valuable, what knowledge gaps remain to support their widescale clinical implementation, and the remaining need for Aβ‐PET in research, clinical trials, and management of AD in clinical practice.

### 1.1 BBMs in AD

A variety of plasma biomarkers have been developed to assess different pathophysiological aspects of AD.^13^ Similar to the development of cerebrospinal fluid (CSF) biomarkers, the first assays encompassed Aβ_42_, Aβ_40_, and phosphorylated tau (p‐tau) 181 to assess the presence of AD pathology. Newer platforms have provided measures of additional p‐tau species, with p‐tau217 emerging as the most robust plasma biomarker of Aβ pathology along the clinical continuum.^2^, ^13^, ^15^, ^16^, ^19^, ^20^, ^21^, ^22^ In contrast, p‐tau205^23^ and particularly the microtubule‐binding region of tau (MTBR‐tau243)^24^, ^25^ seems to be highly specific for tracking tau‐PET burden.

Beyond BBMs of Aβ and tau, markers such as neurofilament light (NfL) and glial fibrillary acidic protein (GFAP) could provide information on other brain changes observed in AD and in other neurodegenerative diseases, representing neurodegeneration and astrocytic activity, respectively.^13^ NfL is considered the most established biomarker for general neurodegeneration,^26^ showing high concentrations in clinical stages of AD and associations with atrophy and brain hypometabolism,^27^ but also increased levels across multiple neurodegenerative conditions and with chronological aging. The astrocytic marker GFAP is associated with Aβ pathology in early disease stages, has prognostic value, and shows a favorable response in the context of immunotherapy‐induced Aβ plaques removal.^28^, ^29^, ^30^, ^31^, ^32^

### 1.2 Assessment of Aβ pathology in clinical populations

In the clinical routine, visual assessment by trained readers of Aβ‐PET images is often employed to assess the presence of Aβ pathology, resulting in in a binary classification of negative or positive for the presence of fibrillary Aβ in the brain.^33^ This approach, yields sensitivity and specificity levels above 90% compared to the gold‐standard, that is, *post mortem* identified pathological burden,^34^, ^35^ and has shown comparable performance for diagnosing AD.^36^, ^37^ Since these initial studies for regulatory approvals over a decade ago, large studies have taken place to understand the clinical utility of the imaging technique, demonstrating an increase in diagnostic confidence and change in patient diagnosis and management after Aβ‐PET.^9^, ^10^, ^38^ Recently, the expert recommendations for clinical use of Aβ‐PET have been updated, suggesting to Aβ‐PET is appropriate in even more clinical scenarios than previously proposed.^39^ Importantly, these recommendations specifically state that only trained specialists should clinically use Aβ‐PET. In the context of ATTs, the use of quantitative assessment to support visual assessments to achieve high certainty of Aβ‐status has been extensively demonstrated.^33^, ^40^, ^41^, ^42^ In particular, the Centiloid metric,^43^ which enables the identification of generalizable cut‐points of Aβ‐positivity and pooling of multi‐center PET data, has received increased interest over the past years.^42^ This metric has been employed as the main inclusion criteria and monitoring tool in the lecanemab/Leqembi® and donanemab/Kisunla® trials.^17^, ^30^ To ensure high certainty of Aβ‐status at the individual level, the AMYPAD consortium recently proposed the use of two cut‐points to rule out (lower threshold) or rule in (higher threshold) Aβ pathology based on Aβ‐PET Centiloid quantification, which has been endorsed by the European Medicines Agency (EMA) with a Biomarker Qualification granted.^42^ The use of quantification might be particularly relevant in early AD or preclinical populations, where Aβ pathology is emerging and less apparent, resulting in lower agreement between readers.^35^

The use of two cut‐points has also been suggested for BBMs, as several studies in clinical populations have illustrated that, while BBMs can differentiate negative versus positive Aβ status reliably in most cognitively impaired patients, even for the most high‐performing BBMs, single cut‐points for classification of Aβ‐status do not yield optimal sensitivity and specificity (84%–90%) against the reference standard Aβ‐PET.^20^, ^44^, ^45^, ^46^ Instead, a two–cut‐point strategy has been proposed, where both a lower probability threshold with 90% sensitivity (to avoid missing detection of patients who are Aβ positive), and a higher probability threshold with 90% specificity (to avoid classifying patients who are Aβ negative as “high risk”) is implemented. This approach results in a so‐called intermediate range, or “gray‐zone”.^13^, ^45^ Algorithms have also been developed that incorporate different BBM together with other easily acquirable factors (e.g., age, apolipoprotein E [*APOE*] genotype, and cognitive screening tests) to improve the overall predictive performance.^3^, ^45^, ^47^ For BBMs, this approach results in > 90% sensitivity and specificity and significantly reduces the further need for additional Aβ biomarker testing (i.e., CSF or PET). However, a considerable fraction of tested individuals (ranging between ∼13% and ∼40.0% in different studies) remain with an unclear BBM‐based Aβ status (i.e., with intermediate BBM values) reflective of an intermediate stage, necessitating confirmatory testing through Aβ‐PET or CSF to ensure accurate detection of Aβ pathology.^3^, ^15^, ^20^, ^45^, ^46^ Given these findings, the recently published Alzheimer's Association Clinical Practice guidelines recommend that  BBM tests with ≥ 90% sensitivity and ≥ 75% specificity can be used as a triaging test, while BBM tests with ≥ 90% sensitivity and specificity can serve as a substitute for amyloid PET imaging or CSF AD biomarker testing in patients with cognitive impairment presenting to specialized care for memory disorders.^48^ It is important to note that the reported sensitivity/specificity of BBMs are against reference standards Aβ‐PET and CSF, which themselves do not achieve full accuracy (i.e., 95% sensitivity and 97% specificity) against the gold‐standard *post mortem* assessments.^49^ As such, a more comprehensive assessment of BBMs performance against *post mortem* validation is needed.

**BOX 1: Open questions of blood biomarkers**

1. How can we further reduce the number of required confirmatory tests (PET or CSF) for the presence of Aβ when using blood biomarkers for initial testing?
2. Can existing blood biomarkers (e.g., different tau phosphorylation species and different Aβ species) be combined in innovative ways to improve measures of quantitative Aβ burden?
3. Which novel blood biomarkers are most likely to fill the most critical current gaps, that is, quantifying Aβ burden before and after Aβ targeting treatment?

### 1.3 BBMs in preclinical populations

Importantly, most two–cut‐point plasma studies have been performed in cognitively impaired populations (mild cognitive impairment [MCI] and/or dementia patients), where the prevalence of Aβ pathology—and thus pretest likelihood—is high.^50^, ^51^ As the field is moving toward both early diagnosis and intervention, understanding the performance of BBM in cognitively unimpaired memory clinic populations, that is, individuals with subjective cognitive decline (SCD), or even the general population, is crucial, especially considering the lower pathological burden and pretest likelihood in this group. Recent work demonstrated that particularly the positive predictive value (PPV) and sensitivity of both an algorithm integrating BBM with demographics, and a stand‐alone test for p‐tau217 occupancy (ratio of phosphorylated to non‐phosphorylated tau‐217 derived from highly accurate mass‐spectrometry assays^52^) were significantly lower for patients with SCD compared to MCI and dementia (∼10%–18% lower in SCD, respectively), though the negative predictive value (NPV) showed generally high performance across diagnostic groups^3^. While the PPV improved with the use of the two cut‐point approach (75% increased to 83%), the overall BBM performance remained significantly lower in the SCD population compared to MCI and dementia, with the two cut‐point approach reaching a PPV of 82% (vs. 95%/99% in MCI/dementia). In addition, 11.5% of SCD subjects fell in the gray‐zone and would require confirmatory testing. While this percentage is relatively low compared to the studies mentioned above due to the use of highly accurate BBMs, it was almost double compared to the MCI and dementia subpopulations (6.5%) in the same study. The first secondary prevention trials in early disease stages are currently ongoing,^53^, ^54^ using Aβ targeting immunotherapies on cognitively unimpaired participants at risk for symptomatic AD. These trials either included plasma Aβ_42/40_ or p‐tau217 as a first step screening tool or as the sole inclusion criteria to determine Aβ‐positive status. Given the lower PPV in presymptomatic populations, relying solely on BBMs risks false positives, unless more conservative thresholds are implemented. Nonetheless, confirmation through Aβ‐PET in this context may be especially important, to avoid unnecessary treatment initiation and associated participant burden and healthcare costs. Taken together, BBMs demonstrate high accuracy in clinical populations, particularly with a two–cut‐point approach and advanced mass spectrometry methods. However, confirmatory Aβ‐PET or CSF testing remains necessary in ∼10%–40% of cases, and potentially more in early/pre‐symptomatic populations depending on the used platforms.

### 1.4 Remaining gaps for routine clinical Aβ status classification

While Aβ‐PET is theoretically a superior marker for assessing Aβ status compared to BBM and has demonstrated excellent comparability in performance across different settings, populations, and radiotracers, its low accessibility, particularly beyond academic sites, and associated costs drastically hamper its widescale implementation in routine clinical use. Significant changes in regulatory policies would be required to improve the scalability and availability of this imaging technique. Within this context, the accessibility of BBMs provides an opportunity for making significant steps to more equitable dementia workups. To achieve this, some remaining gaps need to be addressed. For example, the performance of BBMs in real‐world populations has not been well characterized, and large‐scale studies are needed to determine the performance of BBMs in specific patient subpopulations, underrepresented ethnic groups, and large diverse populations with differing co‐morbidities and concomitant medications to facilitate their proper implementation into clinical routine.^55^ In addition, several studies have demonstrated significant variability in BBM assay performance^56^ with the Alzheimer's Association panel cautioning users, stating that many commercially available BBM tests do not meet their suggested thresholds for clinical use, especially using a single cutoff.^48^ This key element for supporting wide clinical adoption of BBM requires further work to optimize classification strategies and proper teaching efforts to the clinical community at large.

### 1.5 Treatment risk‐stratification

Currently Aβ‐PET or CSF are recommended for therapy initiation.^57^, ^58^ However, considering the high performance of plasma biomarkers in detecting Aβ pathology, they have been widely implemented to screen potentially eligible individuals for participation in clinical trials^53^, ^54^ and in the near future could support identification (or rule out) of AD patients eligible for treatment in clinical practice.^59^ Recent developments and insights from ongoing ATT trials highlight the need for accurate risk‐stratification across disease stages of AD. Results from the donanemab trial demonstrated that patients with high tau burden were less likely to benefit from therapy, while at the same time having an increased risk of side‐effects.^17^ Some initial studies suggest that Aβ‐PET Centiloid quantification could also support the identification of high tau burden, as the prevalence of tau‐PET positivity significantly rises within the 40‐ to 70‐Centiloid range across ROIs reflecting early to established tau burden^60^, ^61^ and no neocortical tau‐PET burden has been observed in cognitively unimpaired individuals with Centiloid levels below 50.^62^ Moreover, at the Clinical Trials on AD (CTAD) 2025 conference, it was demonstrated that this potential high‐benefit group could be accurately selected based on Centiloid quantification, with a reported cut‐point of 60 Centiloid.^62^ Related, higher Aβ‐PET burden was identified as one of the key risk factors for developing Aβ‐related imaging abnormalities (ARIA) after treatment with donanemab,^63^ while lower baseline Centiloid levels were associated with a greater clinical benefit.^64^ As current BBMs (and fluid biomarkers in general) are unable to give accurate predictions of continuous Aβ and tau burden,^20^ they are at the moment suboptimal to support these important steps toward precision medicine. Nonetheless, recent studies illustrate key developments toward this goal, with biomarker combinations of CSF p‐tau species and MTBR‐tau243 77% of Aβ‐PET and 75% of tau‐PET variance could be explained.^65^ In addition, a CSF‐based model, which combined several biomarkers (Aβ42/40, %p‐tau217, %p‐tau205, MTBR‐tau243, and non‐phosphorylated tau) could improve individual‐based disease staging.^66^ Future studies should investigate similar approaches using BBM to optimize their ability to predict continuous PET burden and support risk‐stratification efforts. Within this context, several studies have demonstrated the utility of BBMs, and particularly p‐tau217, to support the identification of specific patient subgroups (e.g., high tau‐PET burden), effectively reducing the number of required PET scans to determine a patients’ risk‐benefit ratio.^20^, ^67^, ^68^

### 1.6 Monitoring treatment effects

The arrival of therapies based on targeting brain Aβ pathology brought not only a need to identify suitable individuals for treatment but also to monitor patients treated with Aβ targeting drugs, to ensure treatment efficacy/target engagement and potentially determine if and when they have reached Aβ clearance. Determining full Aβ clearance may allow discontinuation of treatment for some therapies, reducing patient and facility burden and costs. The TRAILBLAZER‐ALZ2 phase III trial, evaluating donanemab in symptomatic MCI and mild AD patients, already implemented this approach, switching patients to placebo if one PET scan revealed a Centiloid < 11, or if two consecutive PET scans had Aβ levels between 11 and 25 Centiloid (Aβ clearance defined at 24.1 Centiloid).^17^ This approach has been adopted in the prescribing information of the U.S. FDA for Kisunla, which states that treatment effects could be monitored using Aβ‐PET and discontinuation of dosing can be considered.^69^ Importantly, a *post mortem* case‐report of a patient undergoing lecanemab for 1 month supports that signal lowering on the Aβ‐PET scan is reflective of Aβ and phosphorylated tau clearing,^70^ though larger studies are needed to confirm these[Fig alz70763-fig-0001] findings.

Recent results suggest that BBMs are not suitable for tracking treatment efficacy and Aβ removal.^71^ For example, only a moderate correlation (*R *= 0.484) between change in Centiloid and change in plasma p‐tau217 was observed in the donanemab phase III TRAILBALZER‐ALZ trial.^71^ Importantly, the variability in change in plasma p‐tau217 among very high treatment responders, that is, those showing a reduction of ∼100 Centiloid, was large, further highlighting that at the individual level plasma p‐tau217 does not accurately reflect successful Aβ clearance.^71^ Similarly for the lecanemab trial, only numerical improvements in plasma p‐tau181 and Aβ_42/40_ were reported between the placebo and treatment arm.^30^ This limitation of BBMs to function as a therapy monitoring biomarker suggests that currently available BBMs are only indirectly related to actual Aβ burden. Instead, it is possible that they more reflect the underlying metabolic processes (e.g., the development of pathological Aβ strains) that ultimately lead to AD pathology deposits, and those processes likely remain unaffected by the removal of aggregated Aβ. This is supported by experimental animal studies, suggesting that core AD fluid biomarkers are related to the formation of protofibrils rather than deposited Aβ aggregates.^72^ In accordance with this, there is extensive evidence that, while fluid biomarkers and Aβ‐PET have good concordance for binary classification, fluid biomarkers are unable to predict actual Aβ‐PET load, particularly in the positive range of PET tracer uptake.^73^, ^74^, ^75^, ^76^ In addition, all plasma p‐tau species are to some extent associated with both plaques and tangles (although preferentially with plaques in some studies) as based on neuropathology^77^ or PET.^24^ In general, it is poorly understood to what extent plasma biomarker performance observed in natural history studies translates to a treatment scenario. As such, Aβ‐PET will play a crucial role in disease monitoring efforts, particularly in prevention trials, though to what extent Aβ‐PET monitoring will take place routinely in clinical practice is currently unclear, but given the limited accessibility and associated costs of PET imaging, it is unlikely to become a common phenomenon.

### 1.7 Determining treatment intervals

Results from the lecanemab and donanemab trials have demonstrated Aβ re‐accumulation after discontinuation of Aβ targeting treatments, at a similar rate as observed in the general population, that is, (2–3 Centiloid/year).^78^ It may be discussed whether it is beneficial to discontinue Aβ targeting treatments after successful Aβ removal, as proposed in the case of donanemab/Kisunla.^69^ In such a case, guidance is needed on whether and when an individual should resume intervention following successful Aβ clearance. To what extent BBMs could support disease risk‐prediction in this context is unknown. It is possible that currently available BBMs will not have sufficient performance for this task, given unclear relationships between BBMs and pathology burden after treatment. Studies that compare follow‐up Aβ‐PET imaging with longitudinal BBMs in individuals treated with Aβ targeting drugs and who reached successful clearance are needed to investigate this important clinical question.

## 2 CONCLUSIONS

**FIGURE 1 alz70763-fig-0001:**
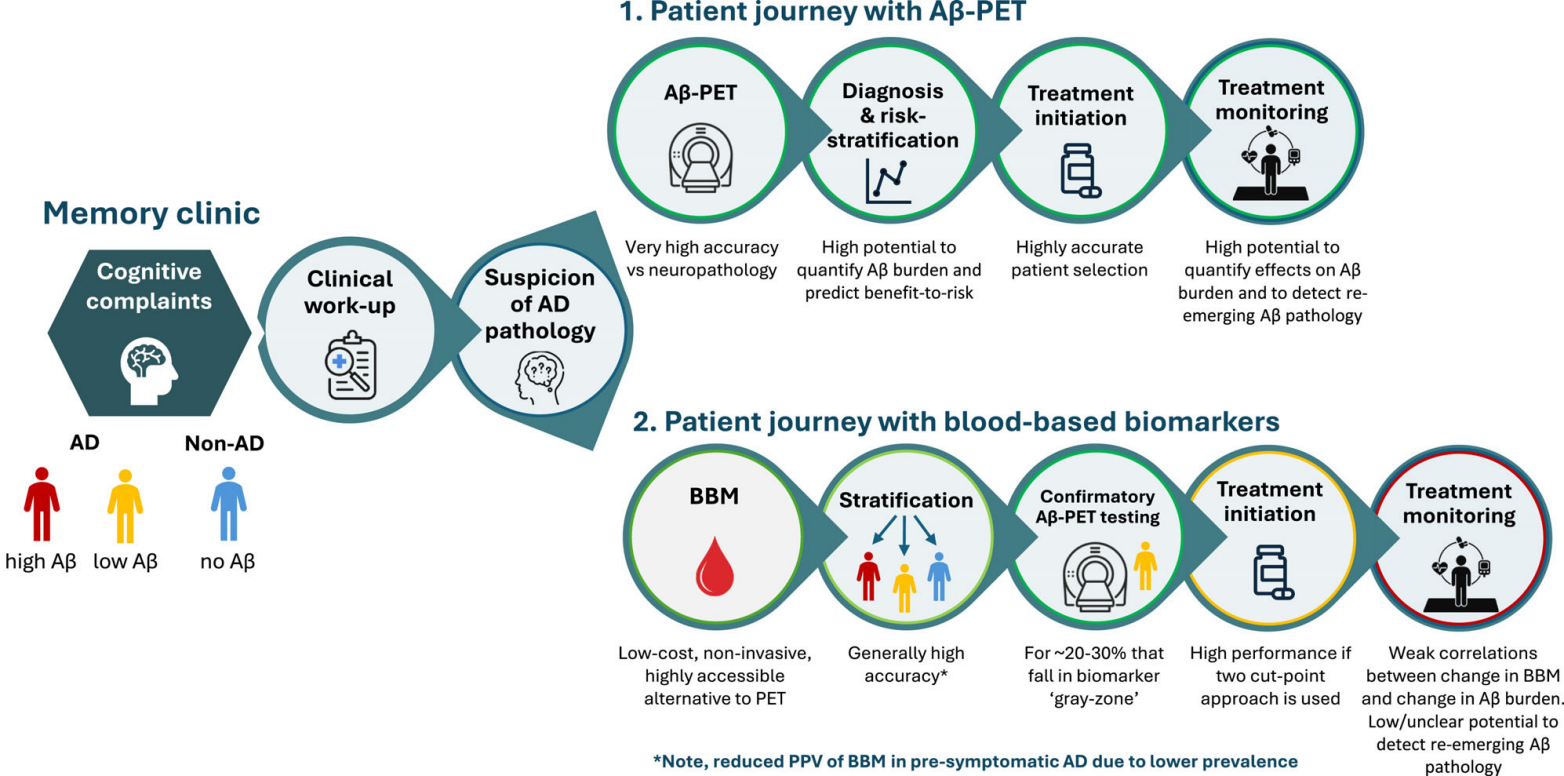
Patient journey based on Aβ‐PET only and including blood biomarkers. Aβ, amyloid‐β; PET, positron emission tomography

Taken together, compared to their more invasive and costly counterpart Aβ‐PET, the arrival of BBMs has enabled wide‐scale assessment of Aβ status in the clinical routine, demonstrating high performance in cognitively impaired patients. However, performance is lower in early/pre‐symptomatic populations due to the inherent lower pre‐test likelihood. The two cut‐point approach to interpret BBMs enables identification of Aβ‐status with high accuracy at the individual level, though confirmatory testing through Aβ‐PET or CSF will be necessary in ∼20% of cases depending on the assay. Recently, guidelines have been proposed on the clinical use of BBMs, a key step toward their implementation in the clinical routine. BBMs are currently not suitable to quantify the overall amount of Aβ pathology (among those who are Aβ positive), but key developments are taking place that suggest that BBMs can support individual staging and risk‐stratification in the near future. There is no support or regulatory endorsement to use BBMs to evaluate effects of Aβ targeting therapy or to guide management of patients after therapy. Here, Aβ‐PET imaging remains a key biomarker (Figure 1).

## AUTHOR CONTRIBUTIONS

Lyduine E. Collij and Niklas Mattsson‐Carlgren drafted the manuscript. Shorena Janelidze, Rik Ossenkoppele, and Oskar Hansson provided feedback on the manuscript.

## CONFLICT OF INTEREST STATEMENT

*L.E.C*. has received speaker/consulting fees from GE Healthcare and Springer Healthcare (paid to institution). *N.M.C*. has received speaker/consulting fees from Biogen, Eli Lilly, Owkin and Merck. *R.O*. has received research funding/support from Avid Radiopharmaceuticals, Janssen Research & Development, Roche, Quanterix and Optina Diagnostics, has given lectures in symposia sponsored by GE Healthcare, received speaker fees from Springer, is an advisory board member for Asceneuron and Johnson & Johnson and a steering committee member for Biogen and Bristol Myers Squibb. All the aforementioned has been paid to his institutions. *S.J*. reports no relevant conflicts of interest. *O.H*. is an employee of Lund University and Eli Lilly. Author disclosures are available in the Supporting Information.

## Supporting information



Supporting Information
